# Rituximab in clinical practice: dosage, drug adherence, Ig levels, infections, and drug antibodies

**DOI:** 10.1007/s10067-017-3848-6

**Published:** 2017-10-04

**Authors:** Jon Thorkell Einarsson, Max Evert, Pierre Geborek, Tore Saxne, Maria Lundgren, Meliha C Kapetanovic

**Affiliations:** 1 Skane University Hospital, Department of Rheumatology, Lund University, Lund, Sweden; 2 Department of Clinical Sciences, Lund University, Section for Rheumatology, Kioskgatan 3, SE-221 85 Lund, Sweden; 3 Skane University Hospital, Department of Laboratory Medicine, Section of Microbiology and Immunology, Lund University, Lund, Sweden

**Keywords:** Adherence to treatment, Anti-rituximab antibodies, Immunoglobulins, Rheumatoid arthritis, Rituximab

## Abstract

The objective of this study is to explore the following: (1) the impact of two different initial doses and cumulative 2-year dose of rituximab (RTX) on drug adherence and predictors of adherence to treatment in rheumatoid arthritis (RA) patients in an observational clinical setting, (2) immunoglobulin levels (IgG/IgM/IgA) during repeated treatment and their relation to infections, and (3) development of anti-rituximab antibodies (ADA). All RA patients receiving RTX from January 2003 to April 2012 at the department were included. The initiating doses were 500 or 1000 mg intravenously days 1 and 15. Drug adherence was estimated using life-table. Baseline predictors of adherence to treatment were analyzed using Cox regression model. Levels of immunoglobulins were measured at treatment initiation and before retreatment. Serum levels of RTX and ADA were measured in 96 patients at 6 months using ELISA. One hundred fifty-three patients were included. Seventy-four (48%) started treatment with 500 and 79 (52%) with 1000 mg. No difference in drug adherence was seen between the different initial or cumulative RTX doses. Methotrexate (MTX) use and low DAS28 at baseline predicted better drug adherence. Ig levels decreased with repeated treatments but low levels were not associated with infections. 11/96 patients had developed ADA at 6 months. Long-term adherence to RTX in RA patient was not influenced by starting- or cumulative 2-year doses. MTX use and low DAS28 at baseline was positively associated with drug adherence. Decreasing Ig levels during treatment were not associated with risk of infections. Development of ADA may influence treatment efficacy and tolerability.

## Introduction

Rheumatoid arthritis (RA) is a chronic inflammatory joint disease with incompletely known pathogenesis. There is substantial evidence suggesting that B cells play an important role in the pathogenesis of RA although the exact mechanisms are not fully elucidated [1–3].

Rituximab (RTX) is a chimeric monoclonal antibody directed against CD20 surface antigens expressed on pre B cells and B cells before their differentiation into plasma cells. RTX causes a selective depletion of CD20 positive B cells but does not affect mature plasma cells and thus does not have an immediate effect on immunoglobulin (Ig) levels [2]. Several studies have shown that RTX improves the signs and symptoms of disease and also retards the structural joint damage in patients with active RA [2, 4–10].

According to the latest EULAR consensus statement, RTX should be considered for RA patients with at least moderate disease activity and who previously had an inadequate response or intolerance to anti-TNF agents [11]. RTX might also be considered in anti-TNF naïve patients in case of contraindications to anti-TNF and with insufficient response to disease modifying anti-rheumatic drugs (DMARDs) such as methotrexate (MTX) [11]. The recommended start dose is 1000 mg given intravenously 2 weeks apart. Repeated treatment could be administered after 24 weeks [12]. Lower RTX doses such as 500 mg intravenously 2 weeks apart have also shown significant treatment efficacy [5]. In a randomized clinical trial including patients with active RA with unsatisfactory response to MTX, no significant difference in ACR20 response at 48 weeks was found between patients who had received initiating treatment with 500 mg twice, 2 weeks apart, followed by retreatment with 500 mg twice after 6 months compared to those receiving corresponding treatment with 1000 mg [9]. A recently published review and meta-analysis of randomized controlled trials comparing low- and high-dose RTX for RA found similar efficacy of both regimes but better safety in patients treated with lower doses [13]. Drug adherence, or length of time on therapy, is an important measure of effectiveness since it is a composite measure for both positive therapeutic benefits as well as negative benefits (adverse event, loss of efficacy) [14].

Retreatment with RTX has been shown to lead to decreasing serum Ig levels which potentially could increase the risk of infections [13–17]. However, a French nationwide register study including more than 1600 patients starting treatment with RTX, identified low IgG levels *before* initiation of treatment as a risk factor of severe infections [14].

The immunogenicity of different biological remedies in terms of development of anti-drug antibodies (ADA) is a well-recognized phenomenon [18–22]. The formation of neutralizing ADA against TNF-inhibitors decreases the levels of active drug and may lead to loss of treatment efficacy [18, 20]. Anti-RTX antibodies have been found both in patients with RA and in patients with other diagnoses but the data on clinical implications of the development of these antibodies are limited [5, 21, 22].

The objective of this study in patients with established RA was to explore the level of drug adherence after two different initiating RTX treatment regimens and the effect of cumulating RTX 2-year dose. We also aimed to identify possible predictors of adherence to therapy. In addition, the objective was to study Ig levels in patients receiving repeated RTX treatment courses and their relation to occurrence of serious infections. Finally, we wanted to study the development of anti-RTX antibodies in a subset of the RA patients.

## Materials and methods

All RA patients starting RTX treatment between January 2003 and April 2012 at Skane University Hospital were included. Data on patient and treatment characteristics were obtained from the South Swedish Arthritis Treatment Register (SSATG) [23–25]. According to previous review, 98% of the RA patients in the register fulfilled the ACR 1987 classification criteria for RA [24]. Briefly, all patients were required to have failed at least one DMARD including MTX before start of any biologic treatment. The decision to initiate biologics and selection of starting dose relied on the treating physician’s judgment and no formal disease activity level was mandatory. RTX could be initiated in biologic naïve patients in case of contraindications to anti-TNF treatment. Patients received either 500 mg 2× or 1000 mg 2× administered by intravenous infusion on days 1 and 15, premedicated with intravenous methylprednisolone. Most patients were scheduled for retreatment at either 6 or 12 months (Table 2). The lower dose (500 mg 2×) was included in local recommendations from 2009. The decision of treatment dose and schedule was made by the treating physician.

Baseline characteristics at initiation of RTX included diagnosis, disease duration, previous and concomitant synthetic DMARDs, prednisolone use, and previous biologic DMARDs. At follow-up visits, the following variables were recorded: health assessment questionnaire (HAQ), patient’s assessment of pain scored on visual analogue scale (VAS pain), general health (VAS global), and 28 tender and swollen joint count. Physician’s global assessment of disease activity and CRP, ESR, and DAS28 ESR was calculated. Patients medical records were searched for serious infections defined as those requiring treatment with IV antibiotics or hospitalization (by JE). Levels of immunoglobulins (IgG, IgM, and IgA) were measured with turbidimetry before initiation of RTX treatment and thereafter prior to RTX retreatment.

In order to validate the RTX dose given and cause of treatment discontinuation, medical records of all patients included in the study were scrutinized by one researcher (ME). RTX discontinuation date was defined as the earliest of one of following: date of death, 12 months after the most recent infusion or date of decision to discontinue RTX, date of adverse event leading to discontinuation, and date of initiation of alternative biologic treatment.

Serum levels of RTX and the presence of ADA at treatment start and at the 6-month follow-up visit were measured in 96 patients who received RTX at the Lund unit. Blood samples were taken immediately before drug administration and analyzed, for both ADA and RTX serum levels, using the Lisa-Tracker Duo Rituximab enzyme-linked immunosorbent assay (ELISA) kit (Theradiag, France). This assay has been developed to reduce low affinity binding of immune complexes or interfering molecules such as rheumatoid factor. The use of specific buffers for both binding and washing steps allows a very efficient capture of free molecules. RTX was considered undetectable for concentrations < 2 μg/mL. The limit of detection of anti-RTX antibodies reported by the manufacturer was 5 ng/mL [26]. Analyses were performed simultaneously for all samples and blinded for clinical data. This study has been reviewed by the regional editorial board.

### Statistical analyses

Mann-Whitney and Chi-square test were used (when appropriate) to compare demographic and treatment characteristics between different starting doses (500 mg, 2×; 1000 mg, 2×). Level of adherence to therapy was measured with drug survival using Kaplan-Meier estimates and COX-regression analysis. Infection-free survival or time to infection was studied using Kaplan-Meier estimates. Possible baseline predictors were studied using COX-regression analysis. Predictors were chosen based on clinical relevance and included sex, age, and disease duration (per year increase), presence of rheumatoid factor (RF), CRP, DAS28-ESR and HAQ scores, concurrent glucocorticoid use (yes/no), concurrent MTX use (yes/no), and calendar year of treatment start (1998–2007). Any collinearity between variables was examined with Spearman correlation testing. A *p* value below 0.05 was considered significant. The results are presented as odds (OR) or hazard ratios (HR) with 95% confidence interval (CI) where appropriate.

## Results

In total, 153 patients (74.5% female) with RA were included. Mean age (SD) and mean disease duration (SD) were 60.7 (11) and 15.8 (12) years, respectively. Percentage of RF and ACPA positive patients were 86 and 82%, respectively. Seventy-four patients (48%) started treatment with 500 mg 2× and 79 patients (52%) with 1000 mg 2×.

Demographics, disease, and treatment characteristics of patients included in the study stratified according to RTX starting dose are given in Table 1.

**Table 1:** Baseline characteristics of patients starting with the two different doses

Rituximab start dose	500 mg 2×	1000 mg 2×	*p* value
Number of patients (%)	74 (48%)	79 (52%)	
Female sex (%)	70	79	0.27
Age (years)	61.0	60.4	0.60
Disease duration (years)	15.9	15.6	0.92
RF positive (%)	88%	84%	0.60
ACPA positive (%)	85%	78%	0.44
ANA positive (%)	45%	58%	0.23
DAS28-ESR (mean; SD)	5.1 (1.5)	5.8 (1.4)	0.008
HAQ (0–3) (mean; SD)	1.3 (0.7)	1.6 (0.6)	0.012
MTX (%)	42%	42%	0.55
MTX dose; mg/week (mean)	15.0	17.2	0.07
Previous DMARDs (mean, range)	4.9 (1–12)	5.8 (1–14)	0.36
Ongoing DMARDs (mean, range)	0.6 (0–3)	0.6 (0–2)	0.19
Prednisolone (yes/no) (%)	70%	75%	0.33
Prednisolone dose; mg/week (mean)	47.4	47.9	0.68
Biologic naïve (%)	20%	14%	0.20
Previous biologics (anti-TNF) (median, range)	1.0 (0–3)	2.0 (0–3)	0.55
CRP mg/L (mean; SD)	15.8 (18)	32.6 (37)	0.001
IgG g/L(mean; SD)	10.2 (2.8)	11.0 (4)	0.39
IgM g/L(mean; SD)	1.5 (0.9)	1.6 (1.3)	0.69
IgA g/L (mean; SD)	3.1 (1.4)	3.1 (1.6)	0.98
History of malignancy	8 (11%)	13 (17%)	0.56

**Fig. 1:**
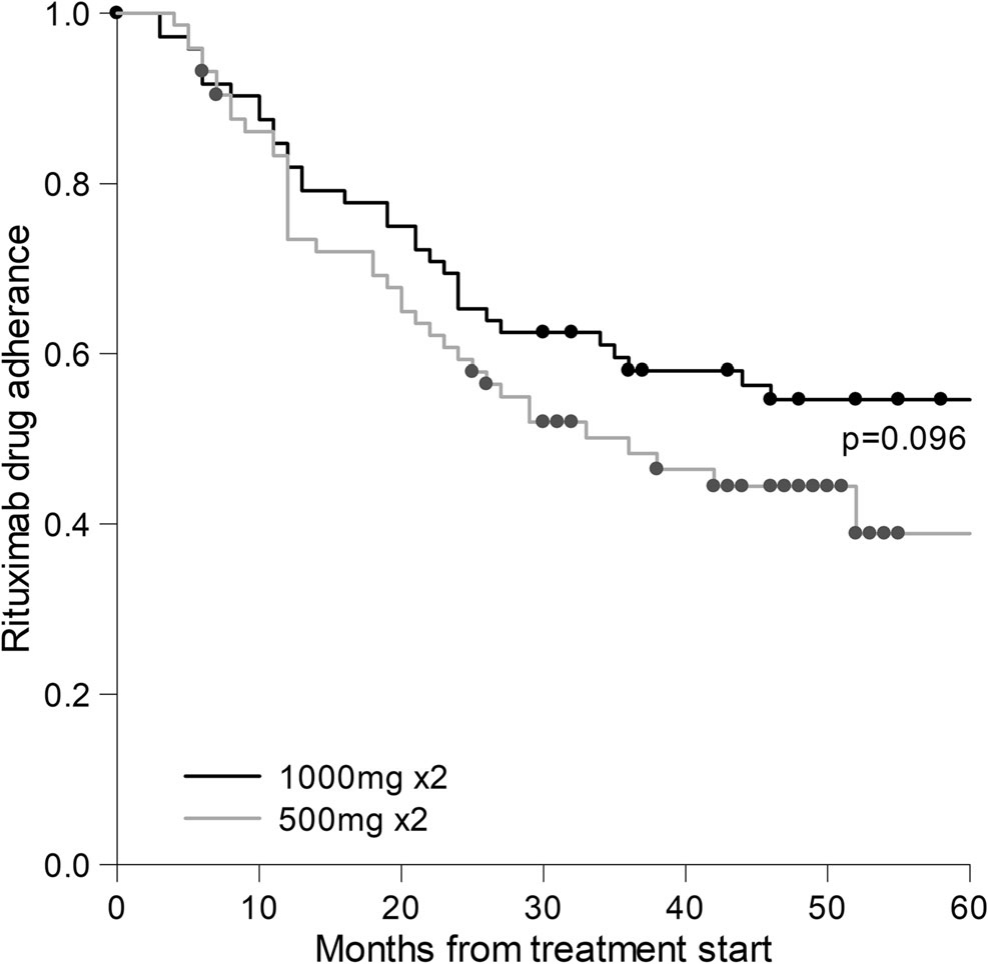
Kaplan-Meier curve showing fraction of patients still on drug at each timepoint during the first 60 months (5 years) after treatment start. The black line represents patients starting on 1000 mg 2× and the gray line 500 mg 2× (log-rank test; *p* = 0.096)

Patients starting with 1000 mg had significantly higher DAS28, HAQ, and CRP at baseline. There were no differences in age, sex, disease duration, concomitant DMARDs, or presence of autoantibodies at baseline between the groups. Numerically but not significantly, more patients not on MTX were taking prednisolone in both treatment groups. Many patients had a history of malignancy, the type was available in 5 of 21 cases, and these were non-Hodgkin and Hodgkin lymphoma, CLL, sarcoma, and lung cancer. (Table 1). Median treatment duration (adherence to therapy) was 44.4 months. A total of 82 (53.6%) patients withdrew from treatment during the first 5 years, most commonly because of failure and adverse event (Table 2). Seven patients were lost to follow up, and the reason for treatment withdrawal is uncertain in 13 patients; none withdrew treatment because of low disease activity. Of the 153 patients, 42 only received the starting dose (Table 2) but not any retreatment. No significant difference was seen for drug adherence when comparing starting doses during the first five treatment years, also when adjusted for baseline characteristics. The cumulative doses of the drug (Table 2) at 2 years did not influence drug adherence. Figure 1 shows RTX drug survival or adherence, in patients with starting doses of 1000 and 500 mg, respectively.

**Table 2:** Treatment characteristics

Rituximab start dose	500 mg 2×	1000 mg 2×	*p value*
Patients receiving only starting dose^a^	16 (22%)	26 (33%)	
Treated every 6 months^b^	53 (72%)	28 (35%)	
Treated every 12 months^b^	5 (7%)	17 (22%)^f^	
Withdrawal from any cause (%)^g^	43 (58%)	39 (49%)	0.29
Withdrawal because of adverse event^g^	11 (15%)	15 (19%)	0.06
Withdrawal because of treatment failure^g^	20 (27%)	16 (20%)	0.44
Methotrexate at 12 months (at baseline)	23% (42%)	27% (42%)	0.58
Prednisolone at 12 months (at baseline)	54% (70%)	66% (75%)	0.36
Cumulative RTX dose at 12 months (g) (mean, SD)^c^	1.84 (1–4)	2.84 (2–6)	< 0.001
Cumulative RTX dose at 24 months (g) (mean, SD)^c^	2.39 (1–6)	3.79 (2–9)	< 0.001
Total exposure (patient-years)^d^	174	278	0.041
ΔIgG (g/L) (SD)^e^	−1.4 (1.3)	−3.9 (2.2)	0.035
ΔIgA (g/L) (SD)^e^	−0.6 (0.6)	−1.5 (0.6)	0.004
ΔIgM (g/L) (SD)^e^	−0.9 (0.5)	−0.9 (0.6)	0.738

^a^Number of patients that received only the starting dose of RTX but were then discontinued for any reason

^b^Number of patients in each group that were planned and received RTX for 6 or 12 months during the first 2 years of treatment

^c^Mean cumulative RTX dose received over the first 12 or 24 months

^d^Total exposure time to RTX in each group

^e^Mean difference between immunoglobulin levels at start and after 60 months

^f^Eight patients (10%) were re-treated after more than 12 months

^g^During the first 5 years

**Fig. 2:**
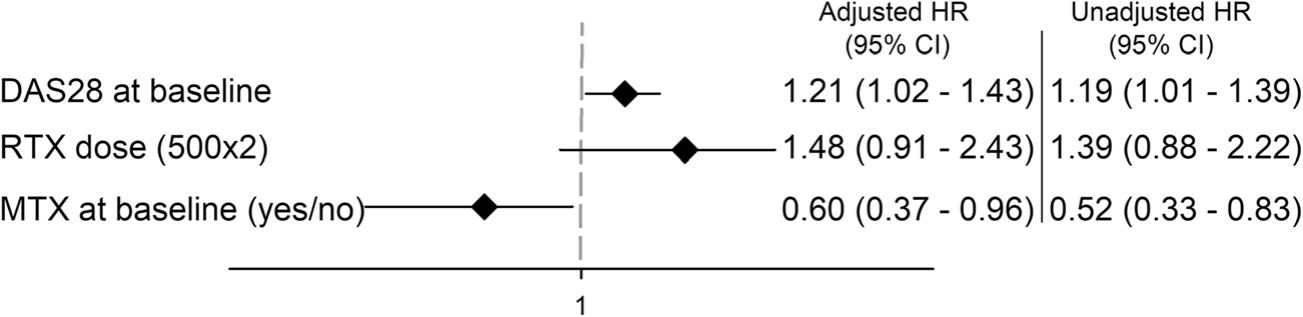
Baseline predictors of rituximab discontinuation during follow-up (adjusted hazard ratios with 95% confidence interval). The figure shows the full adjusted model, a hazard ratio > 1.0 indicates a greater risk of discontinuation. *MTX* methotrexate, *DAS28* disease activity score based on 28-joint count and ESR, *RTX* rituximab

Predictor analysis identified higher disease activity measured by DAS28 at baseline as positively associated, but the concomitant use of MTX at baseline as negatively associated with drug discontinuation (Fig. 2).

**Fig. 3:**
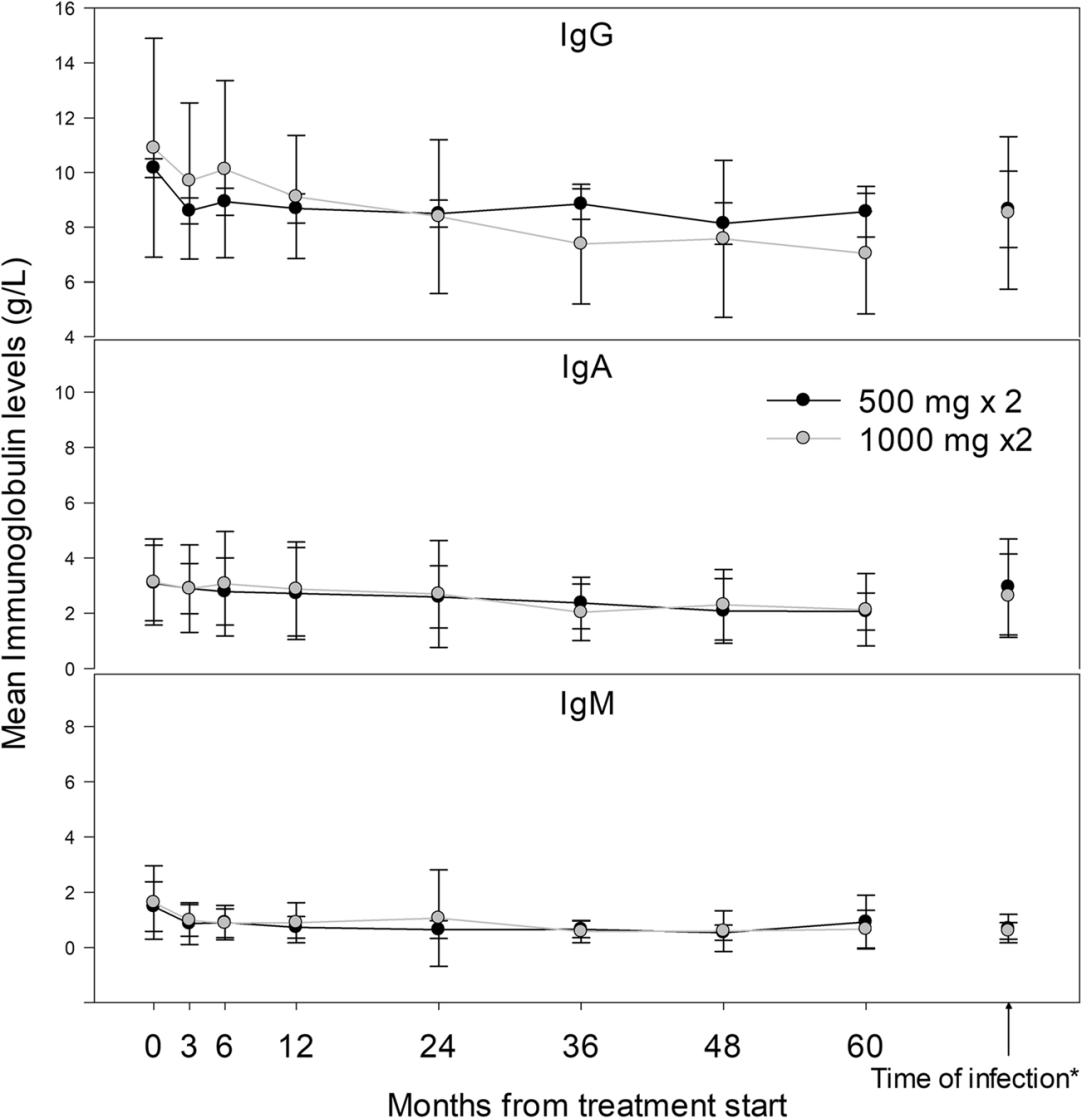
Mean immunoglobulin (Ig) levels with standard deviation over the first 5 years after treatment start. The lines represent different starting dose; 500 mg 2× is shown in black and 1000 mg 2× in gray. The graph on top represents IgG, in the middle IgA, and on the bottom IgM. The first value (month null) is at treatment start. “Time of infection” to the right is the mean Ig level measured in patients with a serious infection

Mean IgG, IgM, and IgA levels at treatment start were 10.5, 1.6, and 3.1 g/L, respectively (Fig. 3). These levels decreased from baseline over time. At baseline, 8.5% of the RTX patients had IgG concentrations below the lower limit of normal (LLN), and 2.4 and 1.2% had IgA and IgM below LLN, respectively. After the start of treatment, 26.1% of patients had IgG levels below LLN, and ≤ 9.7% had IgA or IgM levels below LLN at any time point. At 5 years, patients receiving 1000 mg 2× had a bigger declined from baseline in both IgA and IgG but not IgM, compared to patients receiving 500 mg 2× (Table 2).

**Fig. 4:**
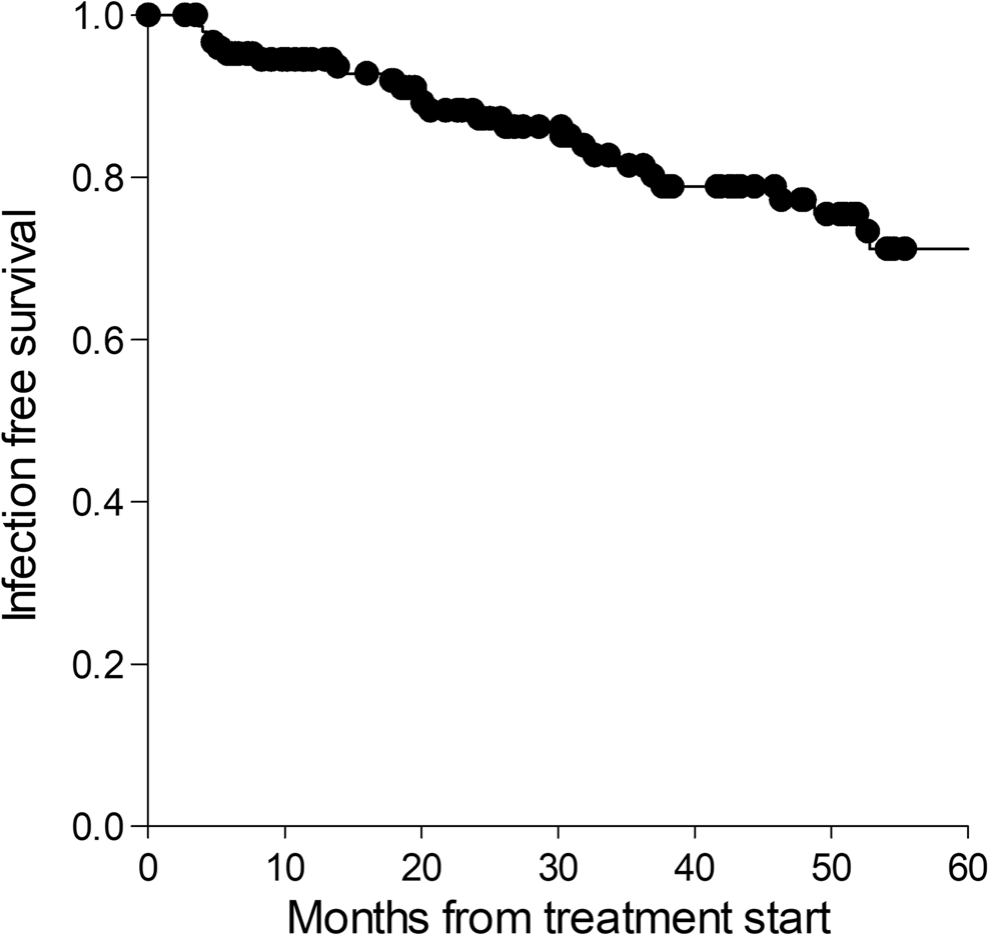
Kaplan-Meier curve showing infection free survival. Serious infections were reported in 36 (23.1%) patients during RTX treatment. The figure also illustrates that there is not an increased risk of serious infection at any specific time point with the events of infection (black dots) spread out over the 60-month follow-up time

Serious infections were reported in 36 (23.1%, 7.9/100) patient-years patients during RTX treatment. No difference in infection rate was seen between different starting doses. The most common infection was respiratory tract infection, affecting 22 patients (14.4%), sepsis (6 patients), cellulitis (2 patients), and herpes zoster (2 patients). The infection rate remained similar over time (Fig. 4). No serious infection was registered during the first 3 months after the start of treatment. Infections were not preceded by Ig-levels below LLN, neither did patients suffering from infection have a significant change in Ig from baseline at any point.

Anti-RTX antibodies were detected in 11/96, i.e., in 11.5% of examined patients at the 6-month follow-up. One additional patient had anti-RTX antibodies before treatment start but no such antibodies at 6 months. Six of the 11 ADA positive patients received 500 mg RTX 2×. Of the ADA positive patients, RTX was given as monotherapy in seven patients and the remaining patients received concomitant MTX (three patients) and hydroxychloroquine (one patient). Two patients with anti-drug antibodies at 6 months reported side effects (skin rush; one of these had no EULAR response); additional four patients reported no EULAR response of treatment at 6-month follow-up, and another two patients had decreasing effect of the treatment compared to the 3 months follow-up visit. The remaining three patients with ADA had good EULAR response at 6 months. There were no significant differences in EULAR response between patients with ADA and those without at 6-month follow-up. The patient with preexisting antibody against RTX had a good EULAR response at 6 months. As expected, serum levels of RTX were very low at the 6-month-follow-up in all 96 patients.

## Discussion

The optimal dosing for RTX in RA has been debated over the years, and the evidence from dose finding studies in RA are still limited. In the present study, we report that in daily clinical practice, RTX starting dose or cumulative dose over 2 years did not seem to influence long term adherence to therapy. Our results are in accordance with previous studies and recent meta-analyses of randomized clinical trials, showing good response to the lower dose and to repeated treatment [7, 9, 13]. These findings are of importance since our study population comprises consecutive patients starting RTX treatment in clinical practice. Many of these patients would not be eligible for a randomized clinical trial.

We also found that concomitant MTX treatment is a predictor for adherence to therapy. One explanation may be that the combined mode of action on both T cells (MTX) and B cells (RTX) has synergistic effect on both treatment and tolerability. In addition, MTX has been shown to reduce development of anti-drug antibodies during treatment with infliximab and could have the same impact on development of anti-RTX antibodies [20, 27]. This being said, the majority of RTX patients have concomitant MTX and those that do not represent a selected group of patients not tolerating MTX.

An interesting finding is that lower DAS28 at initiation of RTX was associated with better adherence to therapy. The patients with high disease activity at the start of RTX have previously failed at least one TNF inhibitor or other biologics (Table 1) and possibly represents a subgroup of “difficult to treat” RA patients not tolerating or not responding to currently available treatment modalities. Long disease duration is another possible explanation since joint swelling and tenderness may be a consequence of irreversible structural damage which also may lead to a higher score on patient reported variables.

The Ig levels have been shown to decrease with repeated RTX treatment, theoretically placing the patients at risk of infections [13, 14, 16, 17]. Our results confirm a decrease in Ig levels after treatment start. However, patients with Ig below lower normal levels did not have more serious infections than those with normal levels. These results are in line with previous findings [14, 16, 28]. One study identified low IgG levels before treatment start as a predictor for serious infections but not the decrease in IgG during treatment [14]. Recently published long-term safety RTX data showed that patients who developed low Ig over time also had more serious infections before Ig levels started to decrease, possibly indicating that these patients have a higher inborn risk of developing serious infections [16].

Our findings that about 11% of patients tested developed anti-drug antibodies are in line with previous studies [5, 20, 21]. Serum samples were collected 6 months after treatment start and immediately before the third RTX infusion. Serum RTX levels at this time point are expected to be low which limits their clinical value for detecting differences between patients with or without ADA. Although we cannon draw any conclusions regarding effect of ADA on treatment efficacy, seven of the 11 patients with ADA reported insufficient response to treatment or decreasing efficacy (*n* = 5) after initially good effect, and two patients experienced infusion reactions which might indicate that development of ADA plays an important role.

Although we consider it important to publish data from daily clinical practice, we acknowledge methodological limitations generated by the non-randomized, open nature of this study [29, 30]. Decision to start treatment or select dose was based solely on clinical practice and experience of treating physician, supported by local, national, and international guidelines. Differences in disease activity and physical function at baseline indicate that the higher RTX dose was reserved for patients with more advanced disease. Although corrected for confounding by indication, channeling- and selection bias or other unmeasured confounders such as comorbidity cannot be completely ruled out. The study can be underpowered to detect any possible differences between different treatment strategies.

In conclusion, starting- or cumulative 2-year doses of RTX used for treatment of RA in this observational clinical setting did not significantly influence the long-term adherence to treatment. Concomitant MTX use and low DAS28 at baseline predicted better drug adherence. In spite of decreasing IgG levels with number of treatment courses, low IgG levels were not associated with increased risk of serious infections. Development of ADA may influence treatment efficacy and tolerability.
